# White common bean extract remodels the gut microbiota and ameliorates type 2 diabetes and its complications: A randomized double-blinded placebo-controlled trial

**DOI:** 10.3389/fendo.2022.999715

**Published:** 2022-10-11

**Authors:** Yuwei Feng, Jie Zhu, Qinyue Wang, Hong Cao, Fang He, Yin Guan, Dan Li, Jiai Yan, Ju Yang, Yanping Xia, Meihua Dong, Feng Hu, Min Cao, Jian Wang, Xiaoying Ding, Yufei Feng, Hong Zou, Ying Han, Su Sun, Jin Zhang, Aijuan Tang, Minhong Jiang, Yu Deng, Jianfen Gao, Yanxin Jia, Wei Zhao, Feng Zhang

**Affiliations:** ^1^ Department of Nutrition, Affiliated Hospital of Jiangnan University, Wuxi, China; ^2^ Clinical Evaluation Center for Functional Food, Affiliated Hospital of Jiangnan University, Wuxi, China; ^3^ Wuxi School of Medicine Jiangnan University, Wuxi, China; ^4^ Department of Infection Control, Affiliated Hospital of Jiangnan University, Wuxi, China; ^5^ Department of Endocrinology, Affiliated Hospital of Jiangnan University, Wuxi, China; ^6^ State Key Laboratory of Food Science and Technology, School of Food Science and Technology, Jiangnan University, Wuxi, China; ^7^ School of Public Health (Shenzhen), Sun Yat-sen University, Guangzhou, China; ^8^ Yinglongqiao Community Health Service Center, Health Commision of Liangxi District, Wuxi, China; ^9^ Department of Health Promotion, Wuxi Center for Disease Control and Prevention, Wuxi, China; ^10^ Department of Functional Examination, Affiliated Hospital of Jiangnan University, Wuxi, China; ^11^ Special Ward, Affiliated Hospital of Jiangnan University, Wuxi, China; ^12^ Department of Urology, Affiliated Hospital of Jiangnan University, Wuxi, China; ^13^ Department of Endocrinology and Metabolism, Shanghai General Hospital, Shanghai Jiao Tong University School of Medicine, Shanghai, China; ^14^ Mashan Community Healthcare Center, Health Commision of Binhu District, Wuxi, China; ^15^ Guangrui and Tongjiang Community Healthcare Center, Health Commision of Liangxi District, Wuxi, China; ^16^ Beidajie Community Healthcare Center, Health Commision of Liangxi District, Wuxi, China; ^17^ Yangming Community Healthcare Center, Health Commision of Liangxi District, Wuxi, Jiangsu, China; ^18^ Shanbei Community Healthcare Center, Health Commision of Liangxi District, Wuxi, China

**Keywords:** white common bean, type 2 diabetes, diabetic complication, gut microbiota, glucose and lipid metabolism

## Abstract

**Objective:**

Excessive carbohydrate intake is a high risk factor for increased morbidity of type 2 diabetes (T2D). A novel regimen for the dietary care of diabetes that consists of a highly active α-amylase inhibitor derived from white common bean extract (WCBE) and sufficient carbohydrates intake was applied to attenuate T2D and its complications. Furthermore, the role of gut microbiota in this remission was also investigated.

**Methods:**

We conducted a 4-month randomized double-blinded placebo-controlled trial. During the intense intervention period, ninety subjects were randomly assigned to the control group (Group C) and WCBE group (Group W). Subjects in Group C were supplemented with 1.5 g of maltodextrin as a placebo. Subjects in Group W took 1.5 g of WCBE half an hour before a meal. Fifty-five participants continued the maintenance intervention receiving the previous dietary intervention whereas less frequent follow-up. The variation in biochemical, vasculopathy and neuropathy indicators and the structure of the fecal microbiota during the intervention was analyzed.

**Result:**

Glucose metabolism and diabetic complications showed superior remission in Group W with a 0.721 ± 0.742% decline of glycosylated hemoglobin after 4 months. The proportion of patients with diabetic peripheral neuropathy (Toronto Clinical Scoring System, TCSS ≥ 6) was significantly lower in Group W than in Group C. Both the left and right sural sensory nerve conduction velocity (SNCV-left sural and SNCV-right sural) slightly decreased in Group C and slightly increased in Group W. Additionally, the abundances of *Bifidobacterium*, *Faecalibacterium* and *Anaerostipes* were higher in Group W, and the abundances of *Weissella*, *Klebsiella*, *Cronobacter* and Enterobacteriaceae_unclassified were lower than those in Group C at month 2. At the end of month 4, *Bifidobacterium* remained more abundant in Group W.

**Conclusion:**

To our knowledge, this is the first report of improvement to diabetes complications by using a dietary supplement in such a short-term period. The enrichment of SCFA-producing bacteria might be responsible for the attenuation of T2D and its complications.

**Clinical trial registration number:**

http://www.chictr.org.cn/edit.aspx?pid=23309&htm=4, identifier ChiCTR-IOR-17013656

## Introduction

Type 2 diabetes (T2D) remains one of the most problematic chronic metabolic disorders. Patients with T2D have a higher risk of macrovascular complications. Intensive glycemic control reduces microvascular complications (1) and exerts a modest improved effect on macrovascular outcomes (2).

Carbohydrates are vital macronutrients that provide primary calories for people, especially those in Asia and Africa. High carbohydrate intake can increase the risk of T2D (3, 4) and total mortality (5). However, the contradiction between the strong desire and rigid control of carbohydrate intake causes a dilemma for patients with T2D. Therefore, a regimen without restricting carbohydrate intake could be a promising antidiabetic approach for patients with T2D.

Carbohydrates can be degraded by α-amylase and α-glucosidase into absorbable monosaccharides in the small intestine. α-amylase can be utilized as a potential target for interventions, and the inhibition of this enzyme may be beneficial for weight loss and glycemic control (6, 7). α-amylase inhibitors are usually purified from white common bean (*Phaseolus vulgaris*). White common bean extract (WCBE) consumption showed a controversial effect on weight loss in animal or human experiment (8–11). In humans, the intake of WCBE was discovered to be able to regulate metabolic diseases, including hyperlipidemia and hyperglycemia (12). Either naturally occurring peptides or hydrolysate fractions from WCBE exerted hypoglycemic activity in rodents (9, 13).

The gut microbiota has attracted considerable attention in recent years as a potentially malleable target for dietary interventions seeking to improve T2D (14). In addition to the direct effect of lowering glucose absorption in the small intestine, supplemental effects can be achieved through regulating the gut microbiota. These effects have deepened our understanding of the hypoglycemic effects of acarbose. ‘Easily digestible’ starches can be transformed by acarbose into ‘slowly digestible’ and ‘long-acting’ carbohydrates (15). Thus, more carbohydrates enter the large intestine (16) and can thereby initiate carbohydrate metabolism by the gut microbiota. Gut bacteria selected by carbohydrates entering the large intestine have capabilities of producing more SCFAs (14, 17). Then, SCFAs can induce intestinal L cells to secrete gut hormones, such as GLP-1 and PYY (14).Because of the similar effect of inhibiting the absorption of carbohydrates by α-amylase and α-glycosidase inhibitors, WCBE may show similar regulatory effects on the gut microbiota. In several animal experiments, WCBE administration has been found to attenuate obesity by modulating the gut microbiota (10, 18). Additionally, correlations have been reported between the regulation of gut microbiota and the improvement of oxidative stress, inflammatory response, and insulin resistance (19, 20), which influence the diabetic complications.

As *Phaseolus vulgaris* was found to exert a weak hypoglycemic effect in patients with T2D, this plant faces an obstacle in its use as an oral antidiabetic agent. Fairly high doses were recommended in clinical studies (21). Extracts of this plant have the potential prospect of modulating postprandial blood glucose levels, but heat treatment during processing aimed at destroying phytagglutinin influences its inhibitory activity. We previously developed a novel extraction method based on ultrahigh pressure (UHP) treatment in WCBE production that can reduce the heat-induced destruction of its inhibitory activities (22). Thus, we used this UHP-treated ready-to-use product of the crude WCBE powder as a food supplement for hypoglycemic purposes in patients with T2D. Furthermore, to date, there have been no reports about the influence of WCBE intervention on diabetic complications.

Here, We conducted a randomized double-blind placebo-controlled trial to explore the effects of WCBE on glucose metabolism and diabetic complications in patients with T2D. Furthermore, we considered the gut microbiota as the breakthrough point and analyzed the underlying important role of the gut microbiota in the improvement of glucose metabolism and diabetic complications by using WCBE.

## Materials and methods

### Study design and participants

The double-blinded, randomized, placebo-controlled 4-month trial was performed from January 2018 to November 2018 in the Third Affiliated Hospital, Nantong University (renamed later as the Affiliated Hospital of Jiangnan University), Wuxi, Jiangsu, China. The trial was approved by the ethical review board of the Third Affiliated Hospital, Nantong University (ID: IEC201711001) and registered on the Chinese Clinical Trial Register as ChiCTR-IOR-17013656. Written informed consent was obtained from all participants before the intervention.

Ninety-six patients with T2D aged 35–75 years were enrolled in this study because they met the following criteria: 6.5%≤ HbA1c ≤13.0%. Besides sulfonylureas and insulins, most hypoglycemic drugs have been reported to be able to modulate gut microbiota (23). Therefore, only patients treated with sulfonylureas or insulin were included. Patients were excluded if they had type 1 diabetes, malignant hypertension, severe cardiac disease, renal failure (eGFR<15), kidney replacement, inflammatory bowel disease, gastrointestinal ulcer, an autoimmune disease, or cancer. Exclusion criteria also included patients receiving medication or surgery for losing weight within 3 months, receiving the administration of antibiotics within one month, receiving gastrointestinal surgery, or during or preparing for pregnancy and lactation. Subjects with poor compliance or protocol violation or unwillingness to continue the clinical trial were asked to withdraw from this study. In the end, ninety participants were eligible and decided to participate in this study.

All subjects were randomly assigned in a 1:2 ratio to the control group (Group C) and WCBE group (Group W). After enrollment, demographic data, including sex, age, disease history, medication and lifestyle, of all subjects were recorded. Anthropometric data, including body mass index (BMI), waist

circumference, hip circumference, blood pressure, and pulse rate, were collected. Sample size was calculated based on the primary outcome (HbA1c), considering a 0.4% difference in HbA1c and a common standard deviation of 0.6% were required to detect a significant improvement in primary outcomes with 95% level and 80% power (24). Considering 10% dropout, withdrawal and non-compliance, a total of 90 subjects were needed in Group C and Group W at a ratio of 1:2.

A simple randomization scheme generated by the computer was created by the trial statistician. The clinical trial was double-blinded because neither the subjects nor researchers knew which group every subject was assigned to, and the grouping of all subjects was unblinded by a statistician after they had completed their experiment. The progression of diabetic complications generally occur over a greater time period than 3 months. Additionally, HbA1c can be used to evaluate the mean level of blood glucose in the recent 2-3 months. In our study, at the end of Month 2, HbA1c was measured after the intense intervention. Parameters of glucose metabolism and diabetic complications were monitored in a part of participant to at Month 4 to determine whether the intervention could ameliorate diabetic complications and maintain the improvement of glucose metabolism. All of the participants received routine dietary guidance for diabetes. Subjects in Group W received 1.5 g WCBE rich in highly active α-amylase inhibitor (raw material preparation technology provided by Suzhou Langbang Nutrition Company and processed by Jiangsu Shouyuan Biotechnology Co. Ltd) half an hour before each meal. Subjects in Group C were given supplementation of 1.5 g maltodextrin as placebo After each participant was assessed for eligibility and assigned to one group, they experienced a 2-week wash-out phase in which their previous diet recipes were changed into dietary regimen for diabetic patient. Afterward, all participants received baseline biochemical and complication indicator measurements. This trial includes two phases. The first phase was a 2-month intense intervention. All participants received daily telephone follow-up, self-monitoring of fasting blood glucose and 2-hour postprandial blood glucose every 3 days, and a weekly face-to-face interview. All subjects only received biochemical examination at the end of this phase. As the progression of diabetic complications generally occur over a greater time period than 3 months, we then randomly selected 22 participants in Group C and 33 participants in Group W who were willing to continue to the next 2-month intervention phase. In this maintenance phase, participants received the intervention with the same dose of WCBE or maltodextrin as the first phase. During the second phase, they only received weekly telephone follow-up. At the end of Month 4, participants received biochemical examination for the third time and complication indicator measurement for the second time.

### The primary and secondary outcomes

The change in the primary outcome, HbA1c, was detected during the intervention. Secondary outcomes included changes in the levels of fasting and postprandial plasma glucose, peripheral neuropathy nerve conduction, endothelial function and fecal microbiota. Outcomes representing lipid metabolism included changes in the levels of total cholesterol (TC), triglycerides (TG), high-density lipoprotein cholesterol (HDL-c) and low-density lipoprotein cholesterol (LDL-c).

### Sample collection and laboratory measurements

Feces and blood were collected from the subjects before the intervention and at the end of month 2 and 4. Venous blood was collected from the subjects after a 10-12 h fast, and the serum was separated for further laboratory measurements. Feces were immediately preserved in a -80°C freezer for further genome sequencing of the gut microbiota.

Clinical laboratory measurements were performed at Wuxi Third People’s Hospital. Blood cell measurements were conducted on an automated hematology analyzer (Sysmex K4500; Sysmex Corporation, Japan). The measurement of biochemical indicators, including fasting blood glucose (FBG), 0.5-hour postprandial blood glucose (0.5 h-PBG), 1-hour postprandial blood glucose (1 h-PBG), 2-hour postprandial blood glucose (2 h-PBG), 3-hour postprandial blood glucose (3 h-PBG), TC, Trig, HDL-c and LDL-c, was performed on an automatic biochemical analyzer (Beckman Coulter au6800; Beckman Corporation, America). Insulin levels were detected on an immunoassay system (Immulite 1000; Siemens Healthcare Diagnostics Inc., Germany). Urine samples collected for urine-microalbumin detection were measured on a biochemical analyzer (Beckman Coulter au5800; Beckman Corporation, America). Fecal samples collected at month 0, 2 and 4 were used to analyze the 16S rRNA gene profile for the gut microbiota.

### Toronto clinical scoring system

The TCSS is based on clinical symptoms, lower limb reflexes and sensory tests, and it was used to evaluate peripheral neuropathy according to a previous study (25). Patients with TCSS scores ≤5 were considered to have no neuropathy, while those with scores ≥6 indicated that they might suffer from diabetic peripheral neuropathy (DPN).

### Nerve conduction study

The NCS was conducted using MedelecSynergy (VIASYS Health care, Philadelphia, PA, USA) based on standardized methodology for NCS (26). Four sensory nerves, including the median, ulnar, sural and superficial peroneal nerves, were tested bilaterally on all the participants. When conducting NCS, it was ensured that the skin temperature was maintained above 32°C over the upper limbs as well as the lower limbs. The parameters recorded in sensory NCS include sensory nerve conduction velocity potential (SNCV) and sensory nerve amplitude.

### Endothelial function analysis

The measurements of ankle-brachial pressure index (ABI) indicate arterial sclerosis and the brachial-ankle pulse wave velocity (baPWV) indicates arterial stiffness and vascular damage. The measurement of these indices were conducted on a BP-203RPE III (Omron Health Co, Kyoto, Japan) according to the instructions for the instrument. The measurements were performed after at least 5 min of resting. Blood pressure of the upper and lower limbs was measured on oscillometric sensors. ABI was calculated bilaterally as the ratio of ankle SBP to arm SBP.

### 16S rRNA gene profiling

Fecal bacterial DNA extraction, NGS library preparation and sequencing were performed at Shanghai Honsun Biological Technology Co., Ltd. (Shanghai, China). Briefly, total DNA was extracted from the stool samples using the E.Z.N.A.^®^ Soil DNA Kit (Omega Biotek, Norcross, GA, U.S.) according to the manufacturer’s instructions. PCR for fecal DNA was performed to amplify the hypervariable V3-V4 region of the bacterial 16S rRNA gene using universal primers 338 F 5’-ACTCCTACGGGAGGCAGCAG-3’ and 806 R 5’-GGACTACHVGGGTWTCTAAT-3’. After purification and quantification, PCR-amplified fragments were used for NGS library construction. 16S rRNA gene sequence tags were generated using Illumina MiSeq PE300 (Illumina, San Diego, CA, USA) and were clustered into operational taxonomic units (OTUs) with a 97% sequence similarity cutoff using the UPARSE v.7.0 platform. The QIIME (version 1.9.1) pipeline was used for 16S rRNA data analysis. Singletons and chimeras were identified and removed before the cluster analysis. Each sequence was taxonomically classified and annotated using Ribosomal Database Project (RDP) Classifier v.2.2 (http://rdp.cme.msu.edu/) against the Silva (SSU138) 16S rRNA database with a confidence threshold of 70%.

The dataset was rarified to 31237 reads for the analysis of α-diversity using four metrics: Shannon, Simpson, Chao and Ace diversity. For beta diversity analysis, principal coordinates analysis (PCoA) was performed on the abundance matrix based on Brary-Curtis distance by R version 3.1.1 (https://cran.r-project.Org/bin/windows/base/old/3.1.1/). The linear discriminant analysis (LDA) effect size (LEfSe) method was applied to determine the OTUs most likely to explain differences between groups at the same time point and between the same group pre- and post-intervention. The pheatmap package (https://cran.r-project.org/src/contrib/Archive/pheatmap/) was used for ecological analysis and heatmap depiction.

### Statistical analysis

Prism 8 (GraphPad Prism, San Diego, CA) statistical software was used to analyze the data. A two-way repeated-measures analysis of variance (ANOVA) with a mixed effects model was used for intra- and intergroup comparisons. Statistical tests were two-sided, and a *P* value < 0.05 was considered to be statistically significant.

## Results

### Baseline characteristics

Ninety-six patients (37 males and 59 females) aged 56-72 years old with T2D who met the inclusion criteria were recruited for this study. Four volunteers who met the exclusion criteria and two volunteers who declined further screening were asked to quit the trial. Thirty subjects were randomly assigned to Group C and sixty subjects to Group W. At baseline, all subjects received measurements of anthropometric, biochemical and complication indicators. At the end of the intense intervention phase, 26 subjects in Group C and 57 subjects in Group W finished the second biochemical examination. We then randomly selected 22 participants in Group C and 33 participants in Group W who were willing to continue the further maintenance phase of intervention. At the end of the second intervention phase, 21 participants in Group C and 31 participants in Group W finished the third biochemical examination and the second complication indicator examination. In this trial, there is no adverse events were reported.The trial profile is shown in **Supplementary Figure S1**.

There was no significant difference in biochemical and diabetic complication indicators in subjects between the two groups at baseline, except for the levels of 0.5 hPBG, Trig, ABI on the left side, median nerve amplitude, and SCV-ulnar nerve (**Table 1**). In addition, there was no change in the participants’ diet structure before and after the intervention (**Supplementary Figure S2**).

**Table 1:** Baseline characteristics of study participants.

Parameters	Group C (n=26)	Group W (n=57)	*P*	
**Basic indicators**				
Age (y)	64.577 ± 1.352	63.912 ± 1.053	0.7142	
Sex (M/F)	8/18	25/32	0.258	
BMI (kg/m^2^)	27.873 ± 0.432	25.086 ± 0.398	0.739	
WHR	0.918 ± 0.009	0.919 ± 0.007	0.923	
HbA1c (%)	7.842 ± 0.151	7.884 ± 0.168	0.864	
**Glucose metabolism indicators**				
FBG (mmol/L)	7.903 ± 0.310	8.464 ± 0.214	0.173	
0.5 h PBG (mmol/L)	10.893 ± 0.470	12.832 ± 0.33	0.001	
1 h PBG (mmol/L)	16.187 ± 0.574	17.079 ± 0.308	0.159	
2 h PBG (mmol/L)	17.920 ± 0.665	19.151 ± 0.453	0.157	
3 h PBG (mmol/L)	16.794 ± 0.799	16.648 ± 0.54	0.883	
**Lipid metabolism indicators**				
TC (mmol/L)	5.031 ± 0.194	4.794 ± 0.123	0.303	
Trig (mmol/L)	2.040 ± 0.265	1.515 ± 0.161	0.034	
HDL (mmol/L)	1.217 ± 0.055	1.344 ± 0.073	0.238	
LDL (mmol/L)	3.050 ± 0.158	2.988 ± 0.109	0.758	
**Biochemical indicators**				
GGT (mmol/L)	27.038 ± 3.254	34.000 ± 5.162	0.384	
UA (μmol/L)	318.038 ± 19.806	311.475 ± 10.572	0.738	
Testosterone (mmol/L)	12.828 ± 1.864	15.003 ± 0.895	0.238	
Leukocytes (×10^9^)	6.677 ± 0.363	6.095 ± 0.186	0.109	
CRP (mmol/L)	1.769 ± 0.279	2.281 ± 0.259	0.199	
mAlb (mg/L)	44.804 ± 25.025	54.621 ± 14.213	0.680	
**Complication indicators**				
TCSS	5.158 ± 0.479	5.115 ± 0.420	0.885	
baPWV-left (cm/s)	1780.471 ± 83.013	1747.115 ± 63.530	0.576	
baPWV-right (cm/s)	1785.882 ± 65.705	1753.154 ± 57.600	0.543	
ABI-left	1.196 ± 0.019	1.132 ± 0.016	0.025	
ABI-right	1.186 ± 0.012	1.158 ± 0.033	0.087	
Ulnar nerve amplitude (mV)	8.811 ± 0.837	7.050 ± 0.596	0.064	
Median nerve amplitude (mV)	14.509 ± 1.930	7.050 ± 0.596	0.0002	
Superficial peroneal nerve amplitude (mV)	12.894 ± 2.449	11.566 ± 1.096	0.8111	
Sural nerve amplitude-left (mV)	7.146 ± 0.927	9.466 ± 0.764	0.0670	
Sural nerve amplitude-right (mV)	6.858 ± 1.020	8.903 ± 0.884	0.1617	
SNCV-ulnar nerve (m/s)	62.028 ± 1.082	56.295 ± 1.223	0.0029	
SNCV- median nerve (m/s)	54.994 ± 2.147	51.714 ± 1.316	0.0859	
SNCV- superficial peroneal nerve (m/s)	56.478 ± 2.494	52.649 ± 1.077	0.1758	
SNCV- left sural nerve (m/s)	56.744 ± 2.094	52.570 ± 1.061	0.0573	
SNCV- right sural nerve (m/s)	54.894 ± 2.261	54.042 ± 1.330	0.4086	

Data are mean ± SEM. UA, uric acid; CRP, C-reactive protein; mAlb, microalbuminuria

### Effects of WCBE on glucose and lipid metabolism

**Figure 1:**
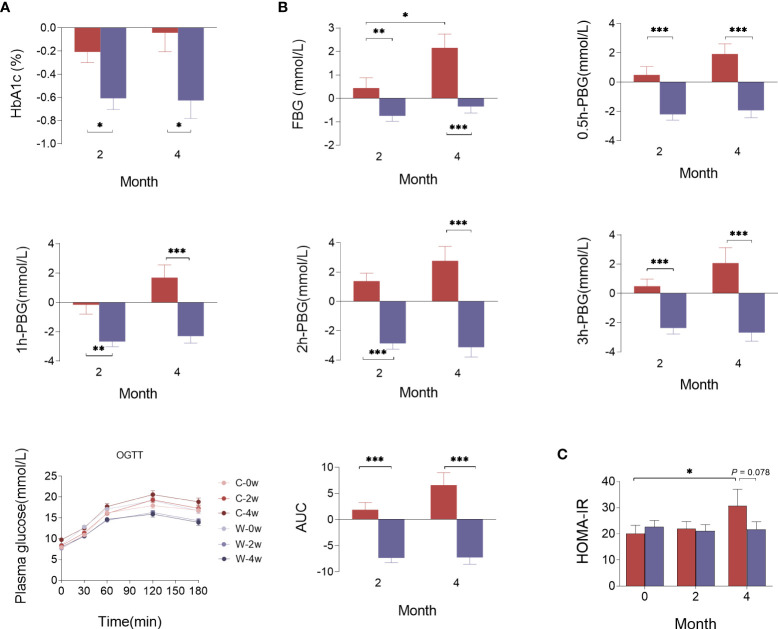
Effects of WCBE on glucose metabolism indices at the end of the 2nd month and 4th month. The change in HbA1c **(A)**, fasting and postprandial blood glucose levels **(B)**, and HOMA-IR **(C)**. *P < 0.05; **P < 0.01; ***P < 0.001.

The trial profile is shown in **Figure 1**. The processed WCBE inhibited the activity of α-amylase to reduce the digestion and absorption of carbohydrates, leading to a decrease in the serum glucose level. HbA1c was the primary outcome. There was a greater reduction in HbA1c levels in Group W than in Group C at the end of the 2-month intense intervention (0.660 ± 0.468% vs. 0.222 ± 0.763%, p<0.05) and at the end of the second 2-month intervention (0.721 ± 0.742% vs. 1.059×10^-8^ ± 0.942%, p<0.05) (**Figure 1A**). In OGTT glucose test, the FBG, 0.5 h PBG, 1 h PBG, 2 h PBG and 3 h PBG in Group W decreased after the 2-month and 4-month intervention, whereas almost all OGTT glucose parameters increased at the end of the 2nd month in Group C and continued to rise at the end of the 4th month. These results indicate an unsatisfactory glycemic control in Group C. Notably, the change in fasting and postprandial glucose levels were significantly different between the two groups at the end of 2nd and 4th month (**Figure 1B**). Accordingly, a significant reduction in the area under the curve (AUC) of glucose levels during the OGTT was observed in those administered WCBE, but this parameter was increased in those administered the placebo (**Figure 1C**). To evaluate the level of insulin resistance, HOMA-IR was calculated according to the fasting blood glucose and insulin levels at month 0, 2 and 4. After the 2-month intervention, HOMA-IR did not change significantly in either group. However, HOMA-IR was significantly increased after the 4-month intervention in Group C but remained steady compared with baseline in Group W. At the end of the 4th month, WCBE tended to lower HOMA-IR levels (*P*=0.078) (**Figure 1C**).

No significant differences were found between the two groups for changes in the lipid metabolism parameters of BMI, waist to hip ratio (WHR), TC, Trig and LDL-c after the 2- and 4-month intervention. However, there was a greater increase in HDL-c after the 2- and 4-month WCBE intervention compared with placebo (*P*<0.05). BMI slightly decreased in both groups at the end of the 2nd month. Although BMI showed a greater increase in both groups at the end of the 4th month compared with the 2nd month, BMI remained relatively steady in Group W. γ-Glutamyl transpeptidase (GGT) was slightly increased in Group C and decreased in Group W, and the change in GGT after 4 months was significantly different between the two groups. Notably, there was a greater enhancement of testosterone in male patients in Group W than in Group C (**Supplementary Figure S3**).

### Effects of WCBE on the progression of diabetic vasculopathy and neuropathy

**Figure 2:**
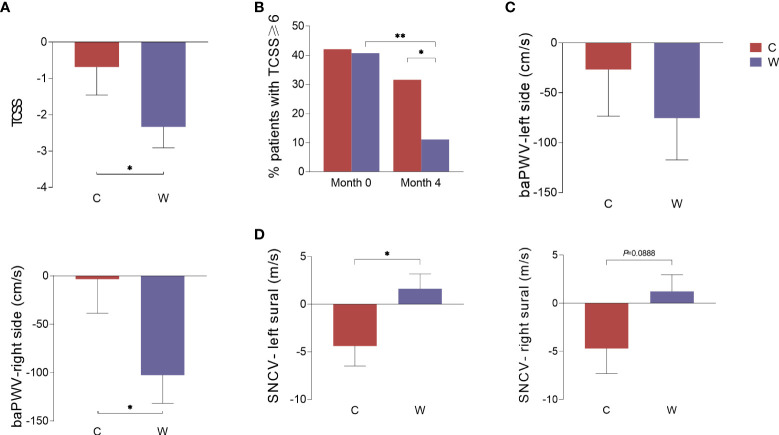
The change in indices representing diabetic complications during the intervention. Data were calculated according to the change in indices in Group C and Group W before and after the 4-month intervention. TCSS **(A)**, the percentage of patients with TCSS≥6 **(B)**, the baPWV on the left and right sides of the body **(C)**, and the conduction velocity of the left and right sural sensory nerves **(D)**. *P<0.05; **P<0.01.

TCSS, NCS and endothelial function analysis were used to assess diabetic vasculopathy and neuropathy. Considering that these diabetes complication indices could not be improved within a short time, we only conducted these measurements before and 4 months after the intervention. The decline in the Toronto score in Group W was greater than that in Group C (*P*<0.05) (**Figure 2A**). We further compared the proportion of patients with TCSS≥6 who could be diagnosed with diabetic peripheral neuropathy (DPN) pre- and post-intervention. We found that this proportion only significantly decreased in Group W after the intervention (*P*<0.01), but no change in this proportion was observed in Group C (*P*>0.05) (**Figure 2B**). Thus, the proportion of patients with TCSS≥6 was significantly lower in Group W than in Group C at the end of 4 months (*P*<0.05). ABI and baPWV are two sensitive markers of arterial stiffness. No difference in the change in ABI was observed between the two groups. As an index reflecting arterial stiffness in the arteries of the lower limbs, the baPWV level on the right side of the body showed greater reduction in Group W than in Group C after the 4-month intervention (*P*<0.05). However, there was no difference in the change of the baPWV level on the left side observed between the two groups (**Figure 2C**).

The NCS is considered the gold standard for the diagnosis of DPN. In this study, both the left and right sural sensory nerve conduction velocity (SNCV-left sural and SNCV-right sural) slightly decreased in Group C and slightly increased in Group W. There was a significant difference in the variation in the SNCV of the left sural nerve between the two groups after the intervention (*P*<0.05), and a slight but not significant difference in the variation in the SNCV of the right sural nerve was observed (*P*=0.0888) (**Figure 2D**). In addition, no difference in the variation in other motor or sensory NCVs were detected between the two groups (**Supplementary Figure 4**).

### Variation of α-diversity of gut microbiota

At baseline, parameters representing community richness and diversity, including the Shannon, Simpson, Chao and Ace index, were not different between participants in Group W and Group C. After 2 months and 4 months of intervention, these α-diversity indices did not change significantly (*P > *0.05, **Supplementary Figure S5**).

### Variation of β-diversity of gut microbiota

**Figure 3:**
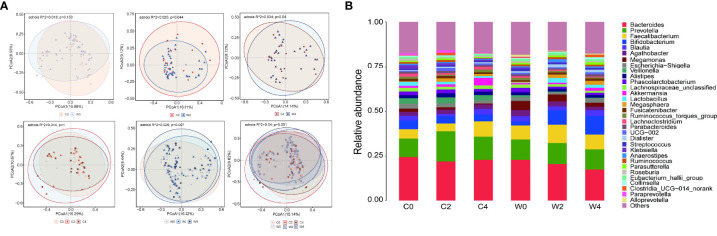
Effect of WCBE treatment on the gut microbiota structure. PcoA of fecal microbiota pre-intervention, 2 months post-intervention and 4 months post-intervention. Based on PERMANOVA statistical analysis, variations during the intervention, *P* = 0.001; C0 vs. W0, *P* > 0.05; C2 vs. W2, *P < *0.05; C4 vs. W4, P < 0.05; variations during the placebo treatment, *P* > 0.05; variations during the WCBE treatment, *P* < 0.01 **(A)**. Microbial community changes at genus level **(B)**.

PCoA based on the Bray–Curtis distance was applied to evaluate the gut microbiota compositional discrimination between subjects in different groups at the same time point and in the same group at different time points. Before the intervention, there was no significant difference in gut microbiota structure between Group C and Group W (PERMANOVA, *P*>0.05). As expected, treatment with the WCBE resulted in a significant difference in the gut microbiota structure compared with Group C at the end of the 2nd month (PERMANOVA, *P*<0.01). At the end of the 4th month of intervention, the gut microbiota structure remained significantly different between Group C and Group W (PERMANOVA, *P<*0.05) (**Figure 3**).

We further compared the gut microbiota structure at pre-intervention with that at 2 and 4 months post-intervention in each group. Significant variations were discovered in Group W after treatment with the α-amylase inhibitor compared with pre-intervention (PERMANOVA, *P<*0.01). However, no variation in the gut microbiota structure could be observed pre- and post-intervention in Group C (PERMANOVA, *P*>0.05) (**Figure 3A**). In the gut microbial community, a higher abundance of *Faecalibacterium* was observed in Group W than in Group C 2 months after the intervention. However, 4 months after the intervention, no difference in the abundance of this genus was discovered between the two groups. The level of *Bifidobacterium* was only increased in Group W 2 and 4 months after the intervention (**Figures 3A, B**).

### Key OTUs that discriminate the differences in the gut microbiota structure after intervention

**Figure 4:**
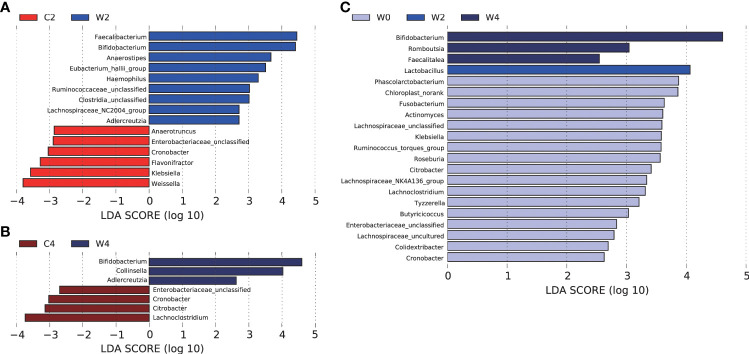
LEfSe analysis of differentially abundant taxa in the genus. Histogram of the LDA scores computed for features differentially abundant between gut microbiota. LEfSe scores can be interpreted as the degree of consistent difference in relative abundance between features in the two classes of analyzed microbial communities. The histogram thus identifies which clades among all those detected as statistically and biologically differential explain the greatest differences between communities.In this study, LDA=2 was used as the cut-off point. Differences in the gut microbiota in Groups C and Group W after 2 months of intervention **(A)**. Differences in the gut microbiota in Groups C and Group W after 4 months of intervention **(B)**.Differences in the gut microbiota between pre- and post-intervention in Group W **(C)**.

LEfSe analysis was performed to identify bacterial taxa with differential abundance among the groups, and only those taxa with a log LDA score >2 were considered. At the end of the 2nd month of intervention, the abundances of *Bifidobacterium*, *Faecalibacterium* and *Anaerostipes* in participants in Group W were higher, whereas *Weissella*, *Klebsiella*, *Cronobacter* and Enterobacteriaceae_unclassified were less abundant than those in Group C (**Figure 4A**). After 4 months of intervention, *Bifidobacterium* remained more abundant in Group W than in Group C. The genus of *Adlercreutzia* also showed higher abundance in participants after the 2- and 4-month α-amylase inhibitor intervention than in those who received placebo treatment. After the 4 months of intervention, *Citrobacter*, *Cronobacter* and Enterobacteriaceae_unclassified were less abundant in Group W. Notably, these less abundant genera after the intervention in Group W all came from the family Enterobacteriaceae, which contains many opportunistic pathogens (**Figure 4B**).

Differential gut bacteria were also screened pre- and post-intervention. In Group W, the abundance of *Lactobacillus* at the end of 2 months of intervention and *Bifidobacterium*, *Romboutsia* and *Faecalitalea* at the end of 4 months of intervention were higher than those before the intervention. The concentrations of *Fusobaterium*, *Roseburia*, *Citrobacter*, *Klebsiella* and Enterobacteriaceae decreased after 4 months of WCBE intervention (**Figure 4C**).

### The dynamic variation of gut bacteria during the intervention

**Figure 5:**
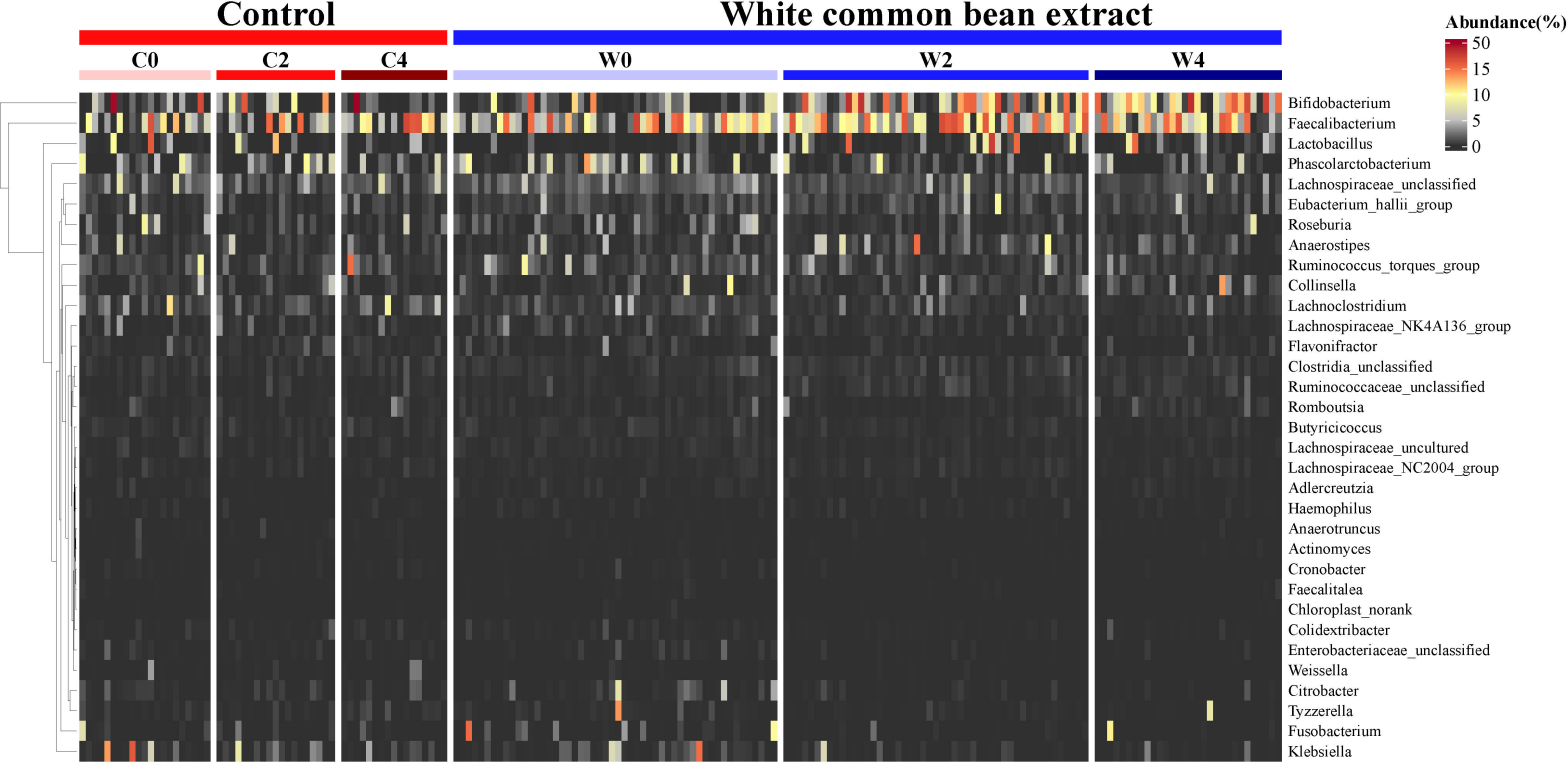
The dynamic change in key OTU abundance during the intervention in both groups. The abundance of the OTUs identified based on LEfSe by comparing the two groups 2 months and 4 months post-intervention and the same group before and after the intervention were used to illustrate the dynamic variation of the key taxa during the α- glycosidase inhibitor intervention.

From the heatmap of the abundance of the key OTUs responsible for the different variations in the gut microbiota structure between the two groups after the intervention, we found that WCBE could enrich SCFA-producing bacteria, such as *Faecalibacterium* and *Bifidobacterium*, at the end of the 2nd month after the intervention. However, after 4 months of the intervention, only *Bifidobacterium* was greatly enriched. The other bacterial genera were not as enriched as those at the end of the 2nd month (**Figure 5**). Moreover, WCBE administration inhibited opportunistic pathogens, such as *Klebsiella*, after 2 and 4 months of intervention, but no significant change was observed in Group C.

## Discussion

In the present study, WCBE that is rich in a highly active α-amylase inhibitor was found to alleviate glucose metabolism dysbiosis and diabetic complication indices. To our knowledge, this is the first time that vasculopathy and neuropathy in patients with T2D have been improved by using a dietary supplement in such a short-term period. Notably, after 2 months of an intense intervention with a WCBE treatment and in the following two-month maintenance period, the improvements to glycemic metabolism were preserved. This finding indicates that WCBE administration might be an ideal choice for the long-term management of glucose homeostasis. Numerous studies have demonstrated a connection between the gut microbiota and the occurrence of T2D. The regulation of the gut microbiota is also associated with improvements of metabolic parameters in patients with T2D (27–29). Since the improvement of some clinical indicators and the gut microbiota structure in Group W was less substantial at the end of the 4th month compared with the 2nd month, we speculate that the gradual diminished therapeutic effect might be related to the gradual diminished improvement of the gut microbiota. More causal relationships still need to be verified by experiments such as gut microbiota transplantation.

To explore the underlying ‘intestinal mechanism’ other than the conventional mechanism of ‘reduced starch absorption’ in glycemic control by the α-amylase inhibitor, we investigated the dynamic variations in the gut microbiota structure during the intervention. Lower α-diversity of the gut microbiota was found in patients with T2D compared with healthy controls (30, 31). In this study, no change in the α-diversity of the gut microbiota could be observed during the intervention. The β-diversity analysis of gut microbiota found that the structure of the gut microbiota was significantly altered by the administration of WCBE and showed significant difference compared with placebo treatment after 2 and 4 months of the intervention. At the end of the 4th month, patients in Group C even showed a trend toward the recovery of their bacterial structure to the pre-intervention status.

In the present study, according to the analysis of the results of LEfSe and heat map, the abundance of *Bifidobacterium*, *Faecalibacterium*, *Anaerostipes* and *Adlercreutzia* after the intense intervention was higher in Group W than in Group C. The higher abundance of *Bifidobacterium* and *Adlercreutzia* in Group W was preserved after the following 2-month maintenance intervention. It was reported that the increase in the abundance of *Bifidobacterium longum* negatively correlates with changes in HbA1c (32). Since many species in *Bifidobacterium* are capable of producing acetic acids with a given amount of carbohydrates, *Bifidobacterium* is often considered an acetic acid-producing genus (14). Acetic acid has been proven to participate in maintaining or improving glucose homeostasis (33). The continuous enrichment of *Bifidobacterium* suggests that this SCFA-producing genus may play an important role in the improvement of T2D during WCBE intervention. In our study, *Faecalibacterium* was more abundant in Group W at month 2. This well-known butyric acid-producing genus has often been inversely correlated with glycometabolism indicators (34). *Anaerostipes*, which has been reported to be capable of converting lactate and acetate to butyrate *via* the acetyl-CoA pathway (35), was higher in Group W at month 2. *Adlercreutzia* is associated with leanness and glucose tolerance (36). We found that the abundance of this genus increased after 2 and 4 months of WCBE intervention. The abundance of *Lactobacillus*, *Bifidobacterium*, *Romboutsia* and *Faecalitalea* significantly increased after the intervention in Group W. *Romboutsia*, a butyrate-producing genus, has been inversely associated with insulin resistance or T2D (37). Faecalitalea has been shown to promote insulin secretion and improve the insulin response (38). Therefore, α-amylase inhibitors can enrich a variety of SCFA producers that can resist systemic chronic inflammation, induce the secretion of beneficial gut hormones, and promote underlying beneficial bacteria that have been reported to be inversely associated with glycometabolism indicators.

Furthermore, the genera of *Klebsiella*, *Cronobacter*, *Citrobacter* and Enterobacteriaceae_unclassified were inhibited by the intake of WCBE. These genera belong to the family Enterobacteriaceae, which contains numerous opportunistic pathogens. *Klebsiella*, a common opportunistic pathogen, was increased in fecal samples from patients with T2D (39). *Cronobacter* can cause bacteremia and sepsis following adherence, invade the mucosa of the gastrointestinal tract and trigger the release of various proinflammatory cytokines and chemokines (40). *Citrobacter rodentium* has been related to defects in mucosal immunity and mucosal barrier integrity, resulting in systemic chronic low-grade inflammation and the further development of diabetes (41). *Weissella* and *Klebsiella* were increased in diabetic mice compared with a control group. They were also decreased after intervention using 1−deoxynojirimycin, which is an α-glucosidase inhibitor. The abundance of these with fasting blood glucose, fasting blood insulin and HOMA-IR (42). *Weissella* has been shown to produce lipoteichoic acids, which are responsible for the induction of host inflammatory responses (43). Thus, WCBE was able to reduce the abundance of opportunistic pathogens with the putative capability of impairing the integrity and immunity of intestinal mucosa and inducing systemic and chronic inflammation in patients with diabetes.

Glycemic control is recognized as an effective means for delaying the progression of diabetic complications. In the present study, WCBE alleviated the glycometabolism indices 2- and 4-months after the intervention. After 4 months of intervention, WCBE improved diabetic angiopathy and peripheral neuropathy, according to the analysis of TCSS, baPWV and left and right sural SNCV. To our knowledge, this is the first report of these effects of an α-amylase inhibitor on diabetic angiopathy and peripheral neuropathy. Accordingly, the gut microbiota was improved after 2 months of WCBE intervention, and these improvements remained after 4 months of intervention.

Recently, in a diabetic mouse model, the genus *Weissella* was inhibited by an extraction from cornuside, which is also a Chinese herbal medicine. The increased abundance of *Weissella confusa* might be closely related to the decreased level of testosterone and reproductive damage (44). *Weissella confusa* MBF8-1 can produce bacteriocin peptides that show spermicidal activity. In our study, WCBE enhanced the level of testosterone, and the genus *Weissella* was less abundant in Group W than in Group C, which is in accordance with a previous report.

Rotundic acid is a constituent in the bark of Ilex rotunda Thunb, and it can downregulate *Klebsiella* abundance. *Klebsiella* is negatively correlated with body weight and positively correlated with parameters of glycolipid metabolism, including glucose, insulin, HOMA-IR, TG, and indicators of cardiovascular function, including ANG-2, MAP, α-HBDH, CK-MB and LDH, in rats (45). In our study, the abundance of *Klebsiella* decreased after the intervention in Group W, which indicates that the inhibition of this genus may participate in the improvement of cardiovascular function. This study offers a novel perspective regarding the relationship among gut microbiota, blood glucose, and diabetic complications, especially the direct effect of the gut microbiota on diabetic complications.

However, there are some limitations in the present study. For example, this study is a randomized double-blind placebo-controlled trial and the sample size is a little small, which may result in the significant differences in levels of some complication indexes. Although the abundance of SCFA-producing genera was increased after the WCBE intervention, the levels of the different SCFAs were not measured. Four months might be too short of a period to obtain a significant improvement in diabetic complications, so significant changes in many indices indicating vasculopathy and neuropathy were difficult to observe in this study. To observe the combinative effects of WCBE on the regulation of glucose metabolic and gut microbiota, the relative small enrolled population may limit the application in a larger population. Later, we will perform further studies to evaluate the clinical effect of WCBE on diabetic patients treated with a variety of hypoglycemic drugs. Metagenomic sequencing would undoubtedly be a better methodology than 16S rRNA gene sequencing, which was used in this study, to analyze the improvements in functions of the gut microbiota and the association between the improvements in the gut microbiota and diabetes and its complications. In addition, whether the improvement of diabetic complications is mainly due to low absorption of carbohydrates or the modulation of the gut microbiota by WCBE needs further investigation.

In conclusion, WCBE, which is a valuable α-amylase inhibitor, could facilitate the improvement of the structure of the gut microbiota, especially the enrichment of SCFA-producing bacteria and inhibition of opportunistic pathogens. This might be a supplemental mechanism by which glycemic metabolism dysbiosis is alleviated in patients with T2D in addition to the direct inhibition of the absorption of saccharides. Furthermore, we speculate that the continuous regulation of the gut microbiota for at least four months by WCBE might be related to the long-term improvement of diabetic complications. This α-amylase inhibitor could be considered a novel prebiotic antidiabetic agent for the regulation of glucose metabolism and gut microbiota homeostasis and may slightly ameliorate diabetic complications in patients with T2D. Notably, this convenient and valuable dietary supplement, WBCE, will meet the strong desire for carbohydrate intake in patients with T2D during long-term glucose control and complication prevention.

## Data availability statement

The date presented in the study are deposited in the NCBI Bio Sample database accession number PRJNA872293.

## Ethics statement

The studies involving human participants were reviewed and approved by Third Affiliated Hospital, Nantong University (Affiliated Hospital of Jiangnan University). The patients/participants provided their written informed consent to participate in this study.

## Author contributions

YuwF, FZ and QW collected, analyzed, and interpreted the data and co-wrote the manuscript. HC collected, analyzed, and interpreted the data. JieZ designed the study, collected, analyzed, and interpreted the data, and co-wrote the manuscript. WZ designed the study and interpreted the data. FaH collected, analyzed, and interpreted the data and co-wrote the manuscript. YG, DL, JuY, YX, YufZ, HZ, SS, AT, MJ, YD, JG, and YJ collected the data and followed-up on the trial. MD designed the study. FHe examined indices of diabetes complications. MC and JW examined indices of diabetes complications and interpreted the data. XD interpreted the data. All authors contributed to the article and approved the submitted version.

## Funding

This work was supported by National Natural Science Foundation of China (grant numbers 81870544, 81870594); the Natural Science Foundation of Jiangsu Province (grant numbers BK20181132, BK20210060, SBK2022023007); Scientific Research Project of Jiangsu Commission of Health (grant number M2021055); Science and Technology Program Project of Jiangsu Market Supervision and Administration (grant number KJ2022028); Jiangsu Scientific Research Project of Elderly Health (grant number LK2021035); Jiangsu Scientific Research Project of Women’s and Children’s Health (grant number F201741); Scientific Research Project of Wuxi Commission of Health (grant numbers ZZ003, Q201762); Wuxi Scientific and Technological Development Project (grant numbers N20192024, N20191001, Y20212001); Translational Medicine Research Program of Wuxi Translational Medicine Center (grant number 2020ZHYB08).

## Conflict of interest

The authors declare that the research was conducted in the absence of any commercial or financial relationships that could be construed as a potential conflict of interest.

## Publisher’s note

All claims expressed in this article are solely those of the authors and do not necessarily represent those of their affiliated organizations, or those of the publisher, the editors and the reviewers. Any product that may be evaluated in this article, or claim that may be made by its manufacturer, is not guaranteed or endorsed by the publisher.

## Abbreviations

WCBE, White common bean extract; HbA1c, Glycosylated hemoglobin; UHP, Ultrahigh pressure; BMI, body mass index; TC, Total cholesterol.; TG, Triglycerides; FBG, Fasting blood glucose; 0.5-hPBG, 0.5-h blood glucose; 1h-PBG, 1-h blood glucose; 2-hPBG, 2-h blood glucose; 3-hPBG, 3-h blood glucose; HDL-c, High-density lipoprotein cholesterol; LDL-c, low-density lipoprotein cholesterol; TCSS, Toronto Clinical Scoring System; DPN, diabetic peripheral neuropathy; NCS, Nerve conduction study; SNCV, Sensory nerve conduction velocity potential; ABI, Ankle-brachial pressure index; baPWV, brachial-ankle pulse wave velocity; RDP, Ribosomal Database Project; PCoA, Principal coordinates analysis.
