# Supplementary material for: White common bean extract remodels the gut microbiota and ameliorates type 2 diabetes and its complications: A randomized double-blinded placebo-controlled trial
Source: Front Endocrinol (Lausanne). 2022 Oct 11;13:999715. doi: 10.3389/fendo.2022.999715 (PMC9594986; doi:10.3389/fendo.2022.999715)
Supplement: Supplementary file 1 [file DataSheet_1.doc]

**
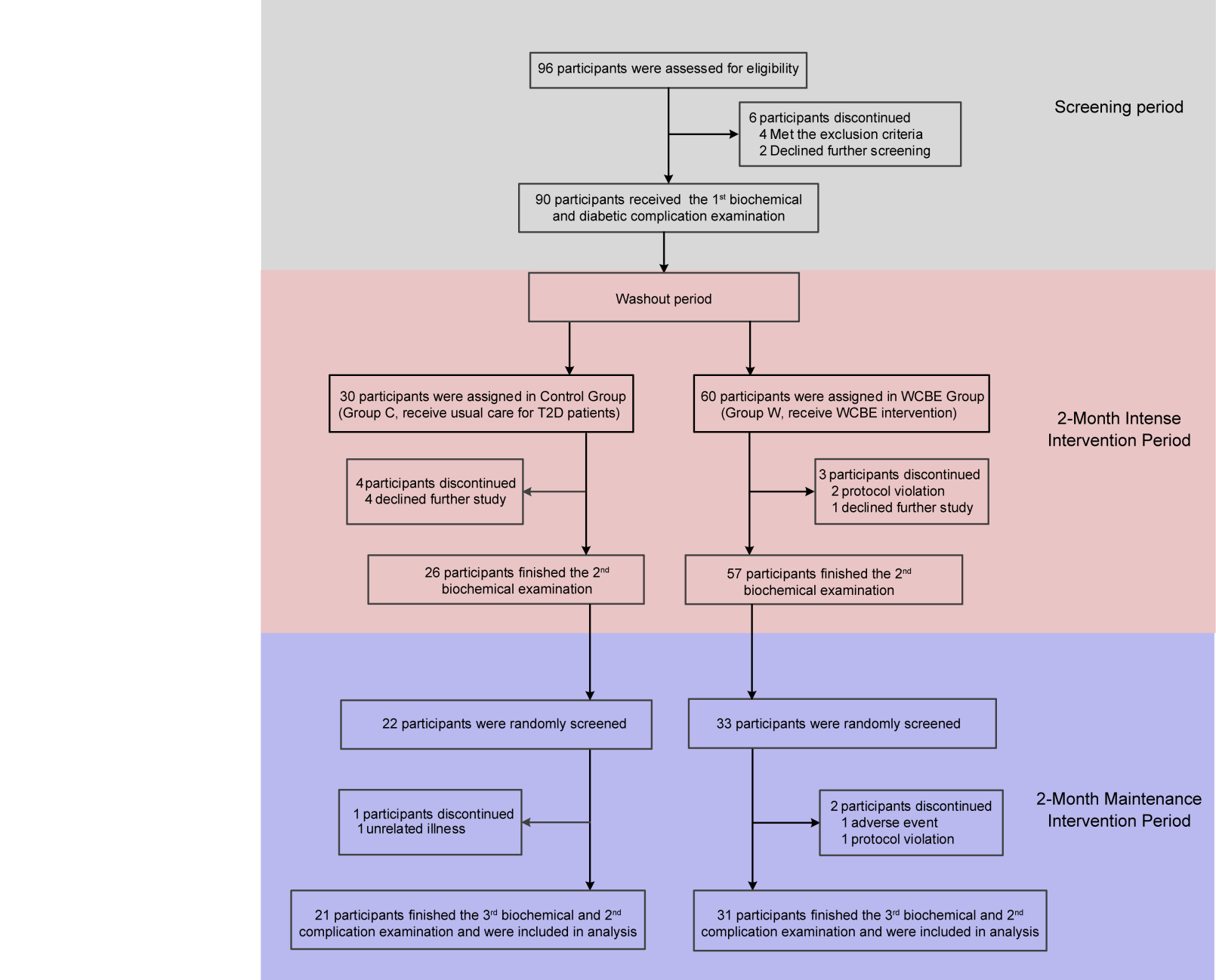
**

**Figure S1 —**Trial profile


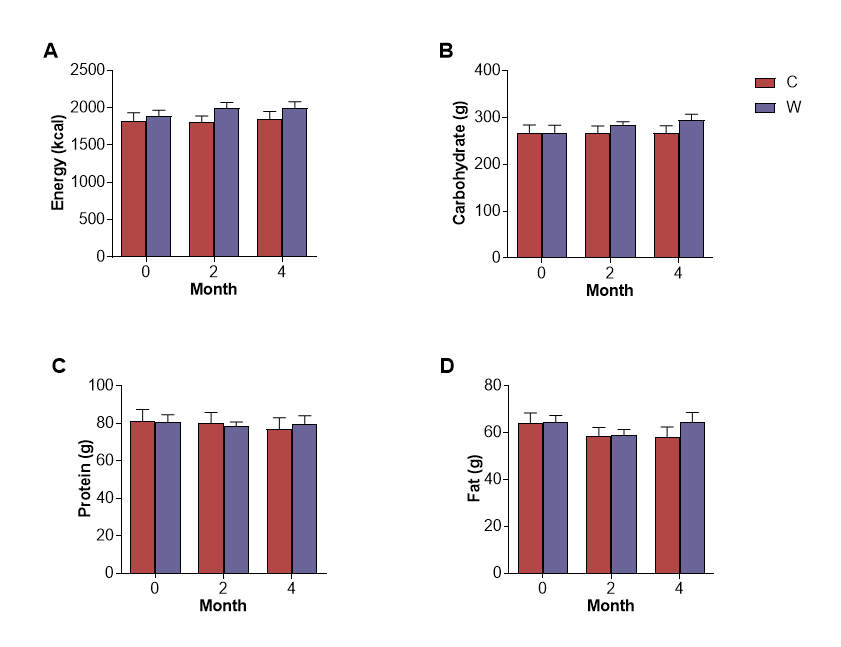


**Figure S2 —** Changes in participants' dietary structure before and after the intervention period. Changes in participant energy before and after the intervention period (A).Changes in participants' carbohydrate intake before and after the intervention period (B).Changes in participants' protein intake before and after the intervention period (C).Changes in participants' fat intake before and after the intervention period (D).


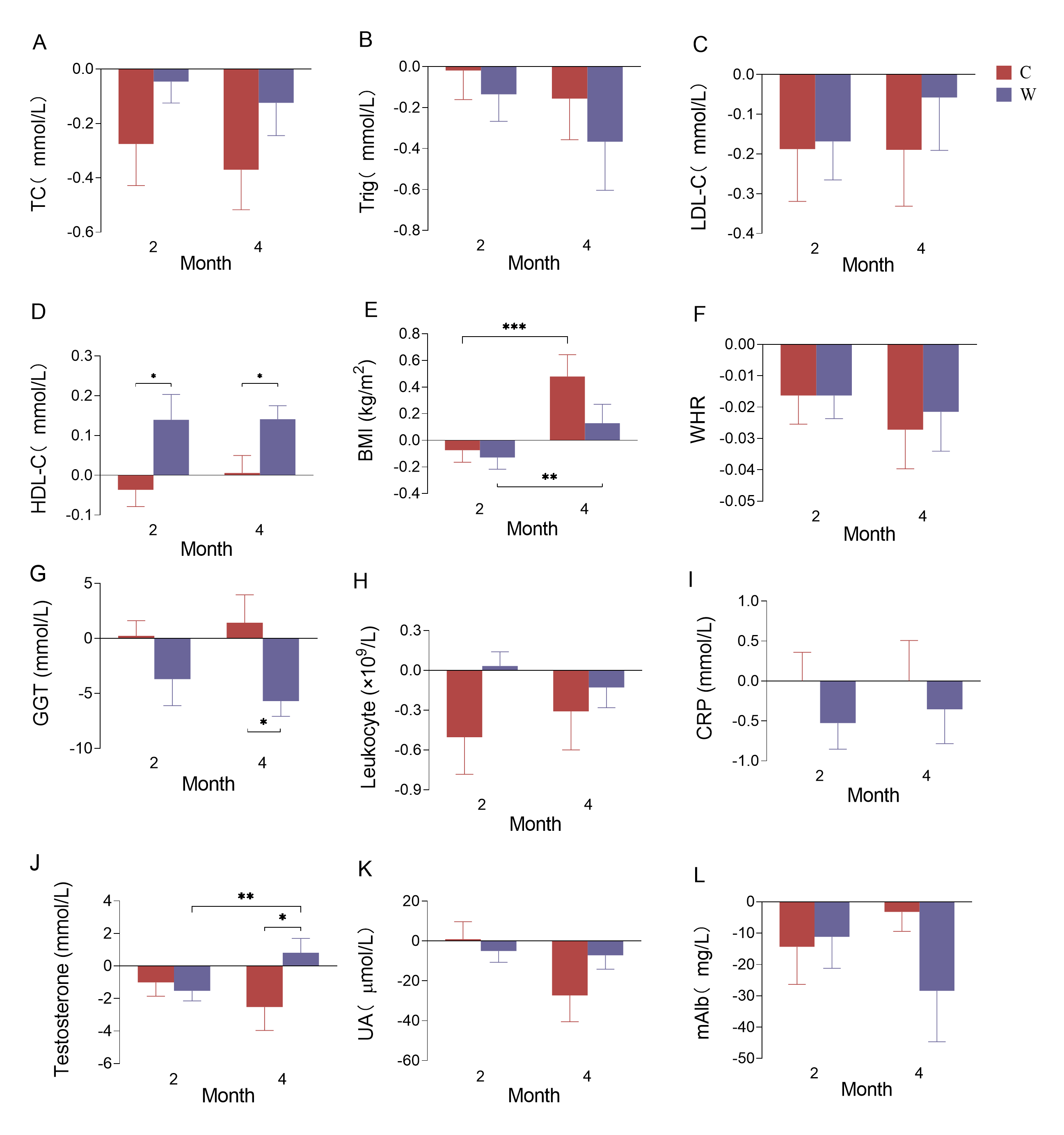


**Figure S3 —** Effects of WCBE on the lipid metabolism (A-G), inflammation (H, I), androgen (J) and kidney function (K, L).


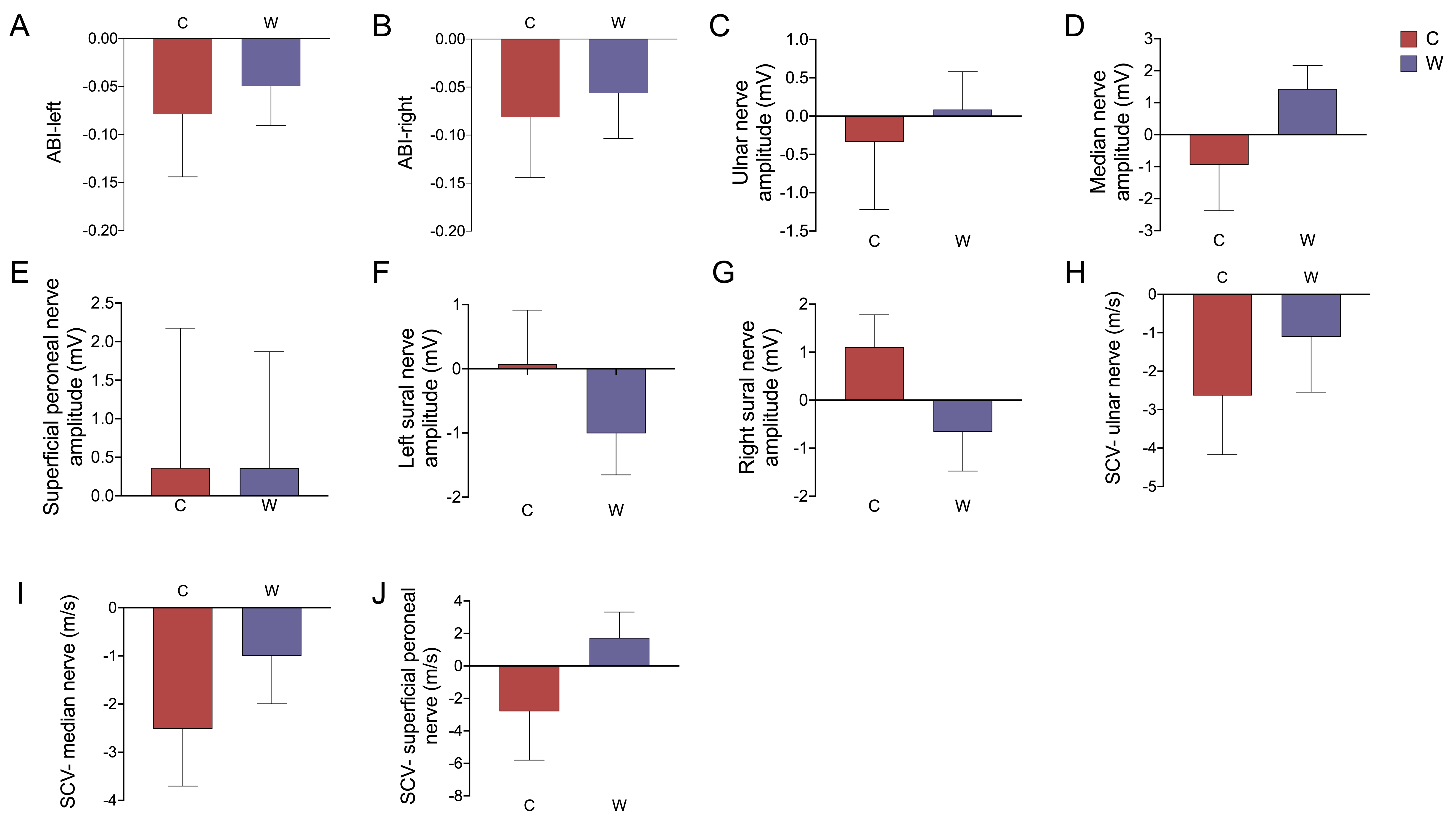


**Figure S4 —** Effects of WCBE on diabetic complications. ABI (A, B), amplitude of the sensory nerve (C-G) and SCV of the sensory nerve (H-J).


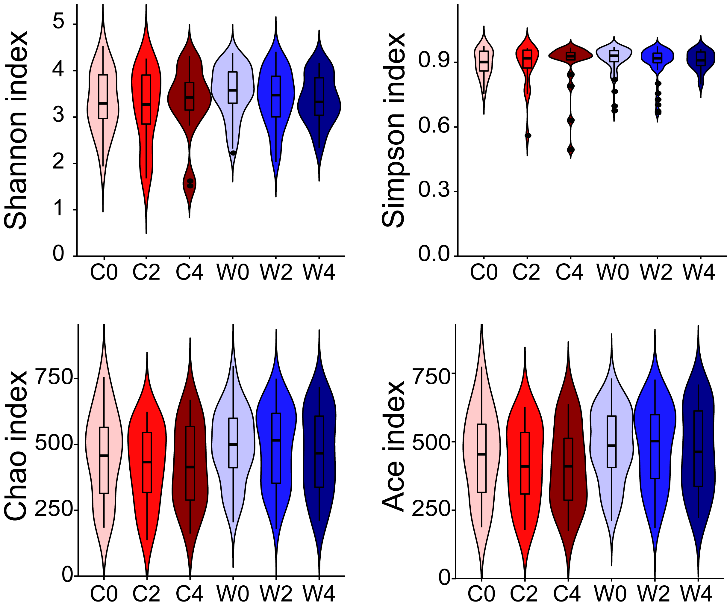


**Figure S5 —** Changes of α-diversity of gut microbiota in two groups during the intervention. C0: Control group before intervention; C2: Control group at the end of 2^nd^ month of intervention; C4: Control group at the end of 4^th^ month of intervention; W0: White Common bean group before intervention; W2: White Common bean group at the end of 2^nd^ month of intervention; W4: White Common bean group at the end of 4^th^ month of intervention.
